# A New Method of Introducing Medicine into the System

**Published:** 1885-11

**Authors:** 


A New Method of Introducing Medicine into the System.

At a meeting of the French Academy of Medicine, held Sept. 22d, M. Brondel read a paper on the introduction of certain medicine into the system by means of electricity. If the electric current is made to pass through a solution of a salt, the salt is decomposed, the metallic base passing to the negative pole, and the acid, or metalloid, to the positive pole. The iodides are easily decomposed by electricity. In order to introduce iodine into the system, a rubber plate, moistened with a solution of iodide of potassium, is placed upon the surface of the body. Over this plate, the negative pole of a battery is applied, while the positive pole is placed upon a part of the body toward which it is desired that the iodine travel. The iodine separates from the potassium, which remains at the negative pole, and passes with great rapidity through the tissues toward the positive pole. This may be demonstrated by testing with a starched paper, which becomes blue. A great number of substances can thus be made to traverse the tissues, and the applications of this discovery are numerous and important. M. Brondel has in this way cured uterine fibroids, a case of perimetritis, rheumatic ovarian neuralgia, and several cases of chronic rheumatism.—*Le Progrès Médical*, Sept. 26, 1885.

Since M. Brondel published his observations on this subject, Dugardin-Beaumetz has made some investigations which, he maintains, show that Brondel's deductions were based upon an error in experimentation. If the hand which carries the positive pole of the battery has not been wet with the iodide solution, the starch paper will remain unaltered, showing that the iodine comes from the operator's hand, and not through the patient's tissues. Brondel has not yet answered these objections.

